# Effect of Paediatric Rehabilitation in Children With Guillain-Barré Syndrome: A Case Series

**DOI:** 10.7759/cureus.59815

**Published:** 2024-05-07

**Authors:** Anushka P Bhagwat, H V Sharath, Pratiksha A Warghat

**Affiliations:** 1 Department of Paediatric Physiotherapy, Ravi Nair Physiotherapy College, Datta Meghe Institute of Higher Education and Research, Wardha, IND

**Keywords:** paediatrics, re-education, weakness, rehabilitation, guillain-barre syndrome

## Abstract

Guillain-Barré syndrome (GBS) is a rare autoimmune disorder impacting the peripheral nervous system, particularly severe in children. This case series assesses the efficacy of paediatric rehabilitation on functional outcomes in paediatric GBS patients. The interventions focused on balance training, strength enhancement, and activities of daily living (ADLs). Four paediatric GBS patients were enrolled, presenting primarily with weakness and fever. Post-rehabilitation, significant enhancements were noted in motor function, ADLs, and quality of life (QoL). This series underscores the favourable impact of paediatric rehabilitation on GBS, advocating for early initiation to improve recovery and enhance QoL. GBS poses significant challenges, particularly in paediatric populations, necessitating comprehensive management strategies. While the syndrome's acute phase is managed medically, rehabilitation plays a pivotal role in optimizing long-term outcomes. This study aims to evaluate the effect of paediatric rehabilitation interventions on functional outcomes in children diagnosed with GBS. The four paediatric patients diagnosed with GBS underwent paediatric rehabilitation, comprising balance training, strength enhancement, and ADL exercises. Functional outcomes, including motor function, ADLs, and QoL, were assessed pre- and post-rehabilitation using standardized measures. The most common presenting symptoms in the paediatric GBS patients were weakness and fever. Following paediatric rehabilitation, significant improvements were observed in specific functional outcomes, including motor function, ADLs, and QoL. These improvements underscore the efficacy of paediatric rehabilitation in enhancing functional recovery and overall well-being in these patients. The findings of this case series emphasize the crucial role of paediatric rehabilitation in managing GBS in children. Early initiation of rehabilitation interventions may facilitate better recovery trajectories and improve long-term outcomes. Comprehensive rehabilitation strategies addressing motor function, ADLs, and QoL are essential components of holistic GBS management in pediatric patients. Pediatric rehabilitation interventions, encompassing balance training, strength enhancement, and ADL exercises, demonstrate significant benefits in improving functional outcomes in children with GBS. Early initiation of rehabilitation interventions is pivotal for enhancing the recovery process and optimizing the QoL in pediatric GBS patients. Further research is warranted to validate these findings and refine rehabilitation protocols for optimal outcomes.

## Introduction

Acute inflammatory demyelinating polyradiculoneuropathy, often known as Guillain-Barré syndrome (GBS) or acute inflammatory demyelinating polyradiculoneuropathy (AIDP), is a multivariate, heterogeneous illness. The hallmark of the traditional presentation is an acute, non-febrile, monophasic post-infectious sickness that presents as ascending weakness and areflexia. It is also possible to see anomalies related to the brainstem, autonomic nervous system, and senses. GBS is now the most prevalent cause of acute motor paralysis in children after poliomyelitis has been eradicated [1]. Acute flaccid paresis in children is most frequently caused by GBS. There are not many reliable, extensive population-based studies on the incidence, risk factors, and early clinical features of paediatric GBS [2].

GBS is the most common cause of acute flaccid paresis in children. Few trustworthy large-scale population-based studies have been conducted on the prevalence, risk factors, and initial clinical characteristics of paediatric GBS [3]. Additionally, 50%-82% of children have been observed to have acute infections prior to the beginning of GBS [4]. Individuals suffering from GBS typically complain of ataxia, weakness, and/or unsteadiness. A characteristic of GBS is weakness. The term progressive ascending flaccid paralysis refers to the weakness that usually begins in the legs and progresses to the arms. This process might take several hours, days, or weeks to complete. Usually, the flaw is symmetrical. Moreover, pain and dysesthesias are reported, especially in young children. For nearly half of the afflicted youngsters, pain may be the first symptom. When a juvenile child presents with chronic and unexplained stomach discomfort, for example, the general character of the symptoms may obscure the true diagnosis. These symptoms frequently start to show up two to four weeks after a sickness or vaccination. Fever, aches in the muscles, diarrhea, or an upper respiratory infection are frequently symptoms of the previous sickness [5,6].

In addition, 10%-15% of children with GBS experience urinary retention early on. Roughly half of juvenile GBS patients may have cranial nerve (CN) involvement and accompanying autonomic dysfunction during the height of their illness, and 10%-12% will need a mechanical ventilator. The most often damaged nerve in patients with CN involvement is the facial nerve, which leads to bilateral facial weakness [5]. Histopathological, there are two primary kinds of GBS peripheral nerve damage: demyelinating forms and axonal-degenerating forms. The disease is more likely to affect motor nerves than sensory ones. Based on a histological and neurophysiological foundation, GBS was separated into four different types in 1995: Miller-Fisher syndrome (MFS), acute motor axonal neuropathy (AMAN), acute motor and sensory axonal neuropathy (AMSAN), and AIDP [7].

The characteristic triad of increasing motor weakness, areflexia, and increased cerebrospinal fluid (CSF) protein without pleocytosis is what defines GBS. In 1859, Landry reported the first contemporary account of a disease that was probably AIDP. The diagnosis of paediatric GBS can be delayed because of its variable presentation. Early admission to the hospital and early treatment are important for decreasing the need for respiratory support and improving the outcome [8]. In 1892, Osler gave a more thorough description of acute febrile polyneuritis. Guillain, Barré, and Strohl originally described the typical CSF finding, albumin cytological dissociation (i.e., the rise of CSF protein with normal CSF cell count) in 1916 and expanded the clinical description even further [9,10]. While there has been much research on the clinical features of GBS in children, there has not been much comparison with adult studies [11]. Sarada et al. discovered that CN palsy was more common in children with GBS and that the condition had an acuter start than in adults [12]. Furthermore, children had a similar rate of respiratory paralysis (40%) and dysautonomia (20%) to adults [13]. Physiotherapy played a central role in the rehabilitation process, focusing on improving muscle strength, range of motion, and mobility. Through a combination of exercises, stretching, and functional training, children regained motor function and achieved milestones in their physical recovery. Occupational therapy interventions target activities of daily living (ADLs), adaptive techniques, and assistive devices to enhance independence and participation in daily life. Speech therapy addressed swallowing difficulties and speech impairments commonly associated with GBS, employing strategies to improve oral motor function and communication skills. Psychological support was integral in helping children cope with the emotional impact of GBS, providing strategies to manage anxiety, frustration, and adjustment to physical limitations.

## Case presentation

Patient information

Case 1

A two-year-old female toddler presented at Acharya Vinobha Bhave Rural Hospital (AVBRH), India, exhibiting fever persisting for the past three days and a gradual onset of weakness in her limbs over the last two days. According to the patient's history, she was in apparent good health three days ago when she developed a low-grade, insidious fever. The initial symptoms were alleviated with medication from a local private hospital. However, on the following day, the child experienced progressive weakness in her lower limbs, leading to her admission to a government hospital in Yavatmal. The private practitioner at the initial hospital suspected GBS, prompting the referral to the government hospital. During the three-day hospitalization, the child's condition further evolved, with the mother observing a change in her voice. Intravenous Immunoglobulin (IVIG) was administered over the course of two days. Due to the unavailability of IVIG at the government hospital, the child was subsequently referred to AVBRH for ongoing management.

Case 2

A seven-year-old male was brought to AVBRH with complaints of weakness in bilateral upper limbs and lower limbs. As per the mother, the child was apparently alright five days back, and then, while playing, the child suddenly fell down while running. Since then, the child complained of lower limb pain. Additionally, he started to complain about her lower limb weakness and pain and finds difficulty getting up and standing. With these complaints, the child was taken to a private hospital and was admitted for three days. There he had episodes of vomiting. As there was no improvement in the condition, they brought the child to AVBRH for further treatment and medical management. Then, there she was diagnosed with GBS. The patient was referred for paediatric physiotherapy for improvement in motor function.

Case 3

A three-year-old male toddler was brought to a hospital with complaints of a sudden onset of difficulty walking and generalized weakness. According to the parents, the child was healthy and had been playing normally until two days ago when they noticed a sudden change in his ability to walk. The parents initially thought it might be a minor injury, but as the day progressed, the child's condition worsened. The family consulted a local paediatrician who, upon examination, noted the absence of any signs of trauma or injury. The child's reflexes were found to be diminished, and there was a noticeable weakness in both lower limbs. The paediatrician suspected GBS and promptly referred the child to the neurology department at AVBRH for further evaluation.

At the hospital, the child underwent a series of tests, including nerve conduction studies and lumbar puncture. The results revealed features consistent with GBS, confirming the diagnosis. The child was admitted for close monitoring, and IVIG therapy was initiated promptly. Over the next few days, the child's weakness progressed to involve the upper limbs, and he developed difficulty swallowing. IVIG therapy was continued, and the child received supportive care, including physical therapy to maintain joint mobility. Despite the challenges posed by respiratory muscle weakness, the child was closely monitored, and non-invasive ventilation was initiated when necessary. The patient was referred for paediatric physiotherapy for improvement in motor function.

Case 4

A two-and-a-half-year-old female toddler was admitted to a hospital with a sudden onset of weakness in her extremities and difficulty in standing. The parents reported that the child had been in good health until a week ago when she developed a mild fever. Initially, they attributed it to a common viral infection and opted for home remedies. However, over the next few days, the child's condition worsened. The parents sought medical attention at a local clinic, where the paediatrician observed bilateral weakness in both the upper and lower limbs. Suspecting GBS, the child was promptly referred to AVBRH for further evaluation and management. Upon admission, the toddler underwent a thorough neurological examination and diagnostic tests, including nerve conduction studies and cerebrospinal fluid analysis.

The results confirmed the diagnosis of GBS. The child was started on IVIG therapy to mitigate the progression of the disease. Upon admission, the toddler underwent a thorough neurological examination and diagnostic tests, including nerve conduction studies and cerebrospinal fluid analysis. The results confirmed the diagnosis of GBS. The child was started on IVIG therapy to mitigate the progression of the disease. Over the course of the next week, the child's muscle weakness continued to evolve, and she developed difficulty in swallowing and speaking. Examination revealed respiratory and motor involvement the patient was referred to the paediatric physiotherapy department.

Clinical findings

Then, paediatric physiotherapeutic assessment was done where the patient was attentive, conscious, and well-oriented to time, place, and person. Additionally, the child attained all the developmental milestones according to the normal age. The reflexes are all intact, and superficial sensations were intact in the bilateral upper limb. The motor examination is depicted in Table 1, which includes muscle tone, according to the tone grading scale, the reflexes, balance, gait, and coordination assessment.

**Table 1:** Motor Examination Pre-rehabilitation Tone assessment (pre-rehabilitation)-1+: decreased response (hypotonia); balance assessment (pre-rehabilitation)- 3/10: moderate impairment with difficulty in maintaining balance; 8/10: slight imbalance; 2/10: severe impairment with difficulty in maintaining balance; 1/10: severe imbalance with difficulty in standing; reflex assessment, absent: 0; present: 2+; gait assessment (pre-rehabilitation)-15/30: unsteady gait with a tendency to stumble; 18/30: slightly altered gait pattern; 12/30: marked unsteadiness with frequent stumbling; 10/30: profound unsteadiness and difficulty in walking; coordination assessment (pre-rehabilitation)-25/40: noticeable difficulties in coordinated movements; 28/40: mild coordination difficulties; 20/40: profound difficulties in coordinated movements; 15/40: severe difficulties in coordinated movements

Tone (Pre-rehabilitation)	Case 1	Case 2	Case 3	Case 4
Upper limb	1+	1+	1+	1+
Lower limb	1+	1+	1+	1+
Reflexes (Pre-rehabilitation)				
Plantar response	0	2+	2+	0
Knee jerk	2+	0	2+	0
Ankle jerk	0	2+	0	0
Biceps jerk	0	2+	2+	2+
Triceps jerk	2+	2+	2+	0
Balance Assessment (Pre-rehabilitation)	3/10	8/10	2/10	1/10
Gait Assessment (Pre-rehabilitation)	15/30	18/30	12/30	10/30
Coordination Assessment (Pre-rehabilitation)	25/40	28/40	20/40	15/40

Intervention

The types of exercise programs and their impact on strengthening and endurance and most exercise programs for the rehabilitation of patients with peripheral neuropathies are symptomatic in nature [14]. Table 2 shows the treatment protocol given to the patient.

**Table 2:** Intervention given to the patients

PROBLEM LIST	Intervention
Knowledge and precautionary care for parents to be taken	This condition requires proper care and precaution as it causes progressive weakness and respiratory and neuromuscular complications. Parents should be included in a rehabilitation program, and all secondary complications should be taken care of. Educate them on performing home exercises and emphasize the importance of consistency and patience in the rehabilitation process.
Respiratory complication	To manage respiratory muscle weakness, strengthen respiratory muscles using a spirometer, consider postural drainage with vibration and percussion techniques, breathing exercises like diaphragmatic breathing and pursed lip breathing, forced expiratory techniques, and play therapy such as bubble blowing.
Reduced range of motion	Stretching Proprioceptive neuromuscular facilitatory techniques (PNF) are included with rhythmic initiation to maintain joint integrity. A task-oriented approach based on the child's daily living activities. Set specific, achievable goals addressing the child's unique challenges and priorities, and gradually increase task difficulty as the child progresses to encourage further improvement. Play-based therapy can incorporate equipment and virtuality devices to promote the development of motor skills in children. Games and toys that encourage targeted activities such as crawling, reaching, and grasping can be chosen based on the child's age and developmental stage. Play therapy activities may include kicking and throwing balls, as well as drawing and clay therapy for fine motor skills development.
Reduced strength	Start with no or low-resistance exercises and gradually increase child tolerance as strength is achieved. Use resistive exercise, functional activities, and playful activities. Strengthen respiratory muscles using a spirometer. Resistive exercise involves using resistive bands and PNF pattern techniques to add resistance to movement. Isometric exercises involve muscle contractions without joint movements, like pulling against a wall or pressing an exercise ball with your leg. Perform functional activities: rise from quadruped to kneeling, half kneeling, sit-to-stand, climb stairs, lift, and carry objects. Use toys, games, and creativity to engage children in enjoyable and age-appropriate strengthening exercises.
Balance, coordination and cognition difficulty	Perform balance and coordination activities like sitting, standing, one-leg standing, walking on different surfaces, tandem walking, alternate block walking, crisscross walking, and balance games. Sitting balance: start with a stool for support, progress to no support, and then to an unstable surface sitting on an exercise ball. Single-leg standing, play therapy (balloon volleyball which can be played in sitting or standing position, which encourages child hand and eye coordination. Hopscotch game activity in which the child will hop over the box or square (this promotes single-leg balance) and figure of 8 walking.

Outcome measures

The outcome measures taken pre- and post-rehabilitation were assessed.

The Ballard score is a comprehensive method used by healthcare professionals to estimate the gestational age of newborn babies. To conduct the assessment, the baby's physical characteristics are carefully examined, including skin texture, the presence of lanugo, plantar creases, breast tissue development, genitalia, ear cartilage stiffness, and overall muscle tone. Each characteristic is assigned a score based on established criteria for both neuromuscular and physical maturity. These scores are then calculated, and a reference chart or table is used to determine the estimated gestational age. However, it is important to remember that the Ballard score provides an estimate and should be interpreted alongside other clinical indicators by trained professionals. This assessment helps healthcare providers monitor the development of newborns and identify any potential complications that may arise. The Denver Developmental Screening Test (DDST) serves as a valuable tool for assessing the developmental progress of children from birth to six years old. Administering the test requires creating a conducive environment and engaging the child in age-appropriate activities across various domains such as personal-social, fine motor-adaptive, language, and gross motor skills. Through interactive play-based tasks, caregivers observe the child's responses and record them meticulously, noting any deviations from expected developmental milestones. Scoring the test involves comparing the child's performance to established norms, identifying areas of strength and potential delays. The results are then interpreted in collaboration with healthcare professionals to determine the need for further evaluation or intervention. Regular administration of the DDST enables early detection of developmental concerns, facilitating timely support and intervention tailored to each child's unique needs.

To access the Peabody Developmental Motor Scales (PDMS), begin by familiarizing yourself with the test's administration procedures and scoring criteria. Ensure you have the necessary equipment and space to conduct the assessment, including items such as balls, pencils, and testing materials provided in the PDMS kit. Next, interact with the child in a comfortable and distraction-free environment, encouraging them to participate in a series of motor tasks that assess their gross and fine motor skills, reflexes, and overall motor development. Administer each task according to the standardized instructions provided in the PDMS manual, carefully observing the child's performance and recording their responses accurately. Once all tasks are completed, score the child's performance based on established criteria, taking note of any delays or discrepancies in their motor development. Interpret the results by comparing the child's scores to age-specific norms provided in the PDMS manual, considering factors such as chronological age and any relevant medical or developmental history. Finally, discuss the findings with appropriate healthcare professionals to determine the need for further evaluation or intervention, ensuring that the child receives appropriate support to address any identified motor difficulties. Regular use of the PDMS allows for ongoing monitoring of motor development and facilitates early intervention to optimize a child's motor skills and overall development. In the Edinburgh Gait Assessment, it is essential to first familiarize oneself with the assessment protocol and procedures outlined in the Edinburgh Visual Gait Score Chart. Ensure you have the necessary equipment, including a camera or smartphone, a gait analysis software or app, and a suitable environment for recording the gait. Position the camera or smartphone at an appropriate angle and distance to capture the entire body during walking. Instruct the individual being assessed to walk back and forth several times, at varying speeds and on different surfaces if possible, while being filmed. Record the gait from multiple angles, including front, side, and rear views, to capture any abnormalities or asymmetries in gait pattern, stride length, foot placement, and overall stability. Once the recording is complete, analyze the footage using the Edinburgh Visual Gait Score Chart, assigning scores to specific gait parameters based on visual observations. Compare the scores to established norms and interpret the findings in the context of the individual's medical history, mobility goals, and functional limitations. Collaborate with relevant healthcare professionals, such as physiotherapists or orthopedic specialists, to develop a comprehensive treatment plan tailored to address any identified gait abnormalities and optimize mobility and function. Regular reassessment using the Edinburgh Gait Assessment allows for monitoring of progress and adjustment of interventions as needed to achieve optimal gait and mobility outcomes.

These are as mentioned in Table 3.

**Table 3:** Outcome measures (pre-rehabilitation) The data have been represented as N; Denver developmental screening test, 85: Slight impairment in cognitive and fine motor skills; 90: Age appropriate; 78: Mild delay; 70: Moderate delay; WeeFIM: NO HELPER 7 Complete Independence; 6 Modified independence; 5: Supervision; 4: Minimal assistance; 3: Moderate assistance; 2: Maximal assistance; 1: Total assistance

Outcome measures (Pre-intervention)	Case 1		Case 2		Case 3		Case 4	
	Pre-treatment	Pre-treatment	Pre-treatment	Pre-treatment	Pre-treatment	Pre-treatment	Pre-treatment	Pre-treatment
Ballard Score	16/18	18/18	16/18	18/18	17/18	18/18	16/18	18/18
Denver developmental screening test	85/98	90/98	90/98	96/98	78/98	90/98	70/98	90/98
WeefIM	03/07	03/07	03/07	03/07	03/07	03/07	03/07	03/07

## Discussion

GBS is a rare autoimmune disorder affecting the peripheral nervous system, leading to muscle weakness and paralysis. While it primarily affects adults, cases in children are not unheard of. Paediatric rehabilitation plays a crucial role in managing GBS in children, focusing on restoring function, mobility, and QoL. This case series examines the impact of paediatric rehabilitation in children with GBS, shedding light on the effectiveness of various interventions. The rehabilitation protocol for children with GBS typically involves a multidisciplinary approach, including physiotherapy, occupational therapy, speech therapy, and psychological support. Each aspect of rehabilitation aims to address different facets of the condition, from physical impairments to cognitive and emotional challenges. Through tailored interventions, therapists work towards maximizing recovery and minimizing long-term disability.

In this case series, children diagnosed with GBS underwent paediatric rehabilitation following different clinical presentations and disease severities. The rehabilitation process commenced promptly after diagnosis, with an individualized plan devised for each patient based on their specific needs and functional deficits. Regular assessments were conducted to track progress and adjust interventions accordingly.

GBS is a rapidly progressing autoimmune disease that damages the peripheral nerve system (PNS). The symptoms, clinical course, and paraclinical findings in childhood GBS have rarely been investigated in non-gait children. Moreover, Pitetti et al. described in their case report an unusual onset of GBS characterized by unilateral peripheral facial paralysis, unilateral lower-limb weakness, and limited outward movement of the eye [15]. In children younger than six years, the manifestations of GBS can include atypical clinical features, such as failure to bear weight, irritability, localized pain with unsteady gait, or even meningism [16]. GBS is an entity that is considered serious and requires multidisciplinary management to prevent complications from occurring and thereby improve the prognosis of patients [17]. In the present case, the healing process was a critical component of the rehabilitation program. The primary areas of concern for physiotherapy therapies are initial acute care, where life must be saved by focusing more on the chest, impairments of strength, impaired coordination, and functional limitations. However, researchers estimate that these deficits account for roughly 40%-50% of life quality reduction expectations [18]. IVIG is delivered to a patient with GBS. The immunological system produces toxins that attack the nerves, resulting in GBS [19]. These are given to assist prevent your nerves from getting harmed by harmful antibodies. IVIG is an immunoglobulin that is inserted directly into a vein [20]. Physiotherapy played a central role in the rehabilitation process, focusing on improving muscle strength, range of motion, and mobility [21].

The results of this case series demonstrate the positive impact of paediatric rehabilitation on children with GBS. Across the cohort, significant improvements were observed in motor function, functional independence, and QoL. Early initiation of rehabilitation and a comprehensive, multidisciplinary approach were associated with better outcomes and shorter recovery times. However, challenges such as variability in disease presentation and individual response to treatment highlight the importance of personalized care in paediatric rehabilitation for GBS. Furthermore, long-term follow-up is essential to monitor for potential complications, recurrence, or residual deficits, emphasizing the need for continuity of care beyond the acute phase.

## Conclusions

In conclusion, paediatric rehabilitation plays a crucial role in optimizing outcomes for children with GBS. Through a holistic approach addressing physical, cognitive, and psychosocial aspects, rehabilitation interventions facilitate recovery, promote independence, and enhance QoL for affected children. Further research and standardized protocols are warranted to refine rehabilitation strategies and improve long-term prognosis in this population. One of the most frequent problems in children is GBS. Early physiotherapy referral, clinical compliance, home program adherence, and family support have all been shown to have a favourable impact on recovery pace. The patient improved from an accurate treatment plan that stressed strength and functional duties and informed the patient's parents about the need for post-discharge care. Although the patient's strength and functioning behaviours were improved, physical therapy was introduced to aid in regaining muscle strength and coordination.
